# Prenatal Stress Impairs Postnatal Learning and Memory Development via Disturbance of the cGMP–PKG Pathway and Oxidative Phosphorylation in the Hippocampus of Rats

**DOI:** 10.3389/fnmol.2020.00158

**Published:** 2020-09-04

**Authors:** Yu-jie Li, Li-ping Yang, Jun-lin Hou, Xin-min Li, Lei Chen, Jiang-hui Zhu, Qi-yang Wang, Gai Li, Pei-yuan Zhao, Xi-hong Liu, Zhan-jiang Shi

**Affiliations:** ^1^Pharmacology Laboratory, School of Basic Medical Medicine, Henan University of Chinese Medicine, Zhengzhou, China; ^2^Department of Integrated Traditional Chinese and Western Medicine, School of Basic Medical Medicine, Henan University of Chinese Medicine, Zhengzhou, China

**Keywords:** prenatal stress, iTRAQ, learning and memory, oxidative phosphorylation, cGMP–PKG pathway

## Abstract

Clinical and animal studies have found that prenatal stress can lead to pathological changes in embryos and fetuses. However, the mechanisms through which this occurs have not been made clear. In the present study, pregnant rats were subjected to chronic psychological stress during gestational days using an improved communication box system, and the changes in behavioral performance and proteins in the hippocampus of offspring were analyzed. It was found that prenatal stress caused postnatal growth retardation and impairment in spatial learning and memory. Furthermore, in isobaric tags for relative and absolute quantitation-based proteomics analyses, 158 significantly differentially expressed proteins (DEPs) were found between the two groups. Further analyses showed that these DEPs are involved in different molecular function categories and participate in several biological processes, such as energy metabolism, learning or memory, and synaptic plasticity. Moreover, the enrichment of pathways showed that the learning and memory impairment was primarily connected with the cyclic guanosine monophosphate–protein kinase G (cGMP–PKG) pathway and oxidative phosphorylation. At the same time, the cGMP level and the expression of PKG protein were significantly decreased, and the neuronal mitochondria appeared to have a swollen and irregular shape in the hippocampus of offspring of stressed rats. These results suggest that the chronic psychological stress that pregnant rats were subjected to during gestational days may have impaired the spatial learning and memory of offspring. This affected the hippocampal oxidative phosphorylation and inhibited the cGMP–PKG pathway.

## Introduction

Clinical and animal studies have shown that prenatal stress can lead to pathological changes in embryos and fetuses. The environmental adversity that is experienced by the mother during pregnancy, whether emotional or physical, affects the growth of the fetus and the physical and mental health of the offspring (Laplante et al., 2004; Soliani et al., 2018). However, the mechanisms through which prenatal stress affects offspring are not yet clear. Many researchers have found that stress may alter the set points of the hypothalamic–pituitary–adrenal and the corticotropin-releasing factor systems and also increase the level of glucocorticoids (Ladd et al., 2000; Welberg and Seckl, 2001). The level of fetal exposure to maternal corticosterone is regulated by the high-affinity, high-efficiency type-2 isoform of the placental enzyme 11β-hydroxysteroid dehydrogenase. However, prolonged exposure to high levels of glucocorticoids in the placenta results in the decreased activity of type-2 isoform 11β-hydroxysteroid dehydrogenase, which leads to a relatively large increase in corticosterone that reaches fetal blood flow (Jensen Pena et al., 2012). At a behavioral level, prenatal stress can enhance emotionality and depression-like behavior, and it can affect spatial learning and memory ability, leading to anxiety-like behavior in offspring (Weinstock, 2001, 2017; Abe et al., 2007; Wang Y. et al., 2015). Prenatal stress can affect the density of the dendritic spine and the dendritic complexity in the hippocampus and the prefrontal cortex of the offspring, and some of these changes are sex specific (Schmidt et al., 2018). At the level of molecular biology, prenatal stress affects the kynurenic acid branch of the kynurenine pathway metabolism in the fetal brain. This may constitute a molecular link between prenatal stress and postnatal brain development (Notarangelo and Schwarcz, 2016). Moreover, prenatal stress-induced depressive-like behavior correlates with hippocampal Avp and Oxt receptor expression, but only in female offspring (Schmidt et al., 2018). In addition, prenatal stress that affects the behavior of offspring might relate to the maternal activation of the renin–angiotensin–aldosterone system (Senko et al., 2017) or through the epigenetic inheritance mechanism of DNA methylation modifications, which may change the expression of genes and the metabolome (Youssef et al., 2018). However, previous studies have not presented a comprehensive understanding of the mechanisms of pathological changes in offspring caused by prenatal stress. A powerful combination of metabonomics and proteomics is often used in pathophysiological studies of depression and spatial memory deficiency (Palmfeldt et al., 2016; Wang et al., 2017; Zhang et al., 2018). Therefore, the results of a comprehensive analysis of metabonomics may be helpful for identifying potential biological relationships between prenatal stress and pathological changes in offspring.

Our previous research has demonstrated that psychological fear stress during pregnancy may affect the cognitive development of offspring, and the mechanism for this might be an imbalance of neurotransmitter secretion in the brain (Xinmin et al., 2017; Liping et al., 2018). However, the precise mechanism has remained unclear. In this study, we examined the hippocampal protein profiles of offspring to determine whether they were affected by prenatal fear stress. The fear stress model (FSM) of pregnant rats was prepared by observing the electrical stimulation of male rats according to the method described in previous studies (Liping et al., 2018; Geng et al., 2019). A proteomic approach based on isobaric tags for relative and absolute quantification (iTRAQ) and a metabolomic approach based on liquid chromatography mass spectrometry (LC–MS) were employed to obtain unbiased profiling data. The STRING database and Cytoscape were used to construct protein–protein interaction networks. Gene Ontology (GO) analyses were performed to analyze the main function of the differentially expressed proteins (DEPs), and the Kyoto Encyclopedia of Genes and Genomes (KEGG) was used to identify the significant pathways of these DEPs. The results help elucidate the complex molecular mechanisms of how prenatal stress can cause the neuronal damage of offspring. They may guide the creation of strategies for the diagnosis and the prevention of diseases caused by prenatal psychological stress.

## Materials and Methods

### Animal Care and Ethics Statement

A total of 65 (40 female) adult Wistar rats [specific-pathogen-free grade; age, 11 weeks; weight, 170–210 g; Shandong Lu Kang Medical Limited by Share Ltd., Shandong, China; license number: SCXK (Lu) 2014–0001] were used in the present study. All the rats were housed in standard laboratory conditions (21 ± 1°C, 55 ± 5% relative humidity, 12/12 h light/dark cycle) and had *ad libitum* access to food and water. The study protocol was approved by the Ethics Committee of Henan University of Chinese Medicine (Henan, China). All the animal treatments were performed according to the National Institutes of Health guidelines (Clark et al., 1997).

### Animal Treatment

#### Experimental Protocol

All rats were housed for 7 days to acclimate them to the environment. Body weight and scores on the Sucrose Preference Test (SPT) (Wu et al., 2016) and the Open-Field Test (OFT) (Smart and Dobbing, 1971) were measured for the rats to ensure the consistency of the research objects and to minimize individual differences. Two outliers were screened using a box plot and were removed. The male rats were measured as well, and one outlier was restricted from mating but not removed. Later, it was used for electrical stimulation tests. When presenting proestrus or estrus, the rats were placed together in a 2:1 ratio of females to males (three rats per box). Mating and gestation were estimated by the presence of a vaginal plug. If it was not found, a vaginal smear was used to examine the presence of sperm. Mating was allowed to continue, and the rats were checked again on the following day. If no vaginal plug or sperm was found, the same procedures were followed for 3 days. After mating, the pregnant rats (those for which a vaginal plug or sperm was found) were randomly divided into the FSM group and the normal control (NC) group, with 15 rats per group. The pregnant rats from the FSM group observed the electrical stimulation of male rats for 20 days, using an improved communication box system, according to previously described methods (Xinmin et al., 2017; Liping et al., 2018; Geng et al., 2019). After this treatment, behavioral changes in the pregnant rats (and their offspring after birth) were observed (more details below). Eventually, the offspring were sacrificed and their hippocampi were excised to examine the relationship between maternal stress during gestational days and brain injury in offspring.

The behavioral assessment of pregnant rats proceeded as follows. During modeling, all the rats were regularly fed, and the consumption of water and food in both groups was monitored daily. The pregnancies lasted for about 21 days. At the end of each gestational week, the scores in the SPT, OFT, and Tail Suspension Test (TST) (Takeuchi et al., 2003; Wu et al., 2016) were observed to evaluate the anxiety of pregnant rats.

The behavioral assessment of the offspring (Smart and Dobbing, 1971; Chiavegatto et al., 1997; Chen et al., 2012; Ivani et al., 2016) was conducted as follows. The number, the weights, and the changes in weight of the pups for each dam were recorded and analyzed statistically. The physical development and the maturation of the pups were evaluated using the methodology proposed by Smart and Dobbing (1971), Chiavegatto et al. (1997), Chen et al. (2012), and Ivani et al. (2016). First, the dates on which surface righting reflexes occurred were recorded (Ivani et al., 2016), with the test performed during postnatal days (PNDs) 4–21, by gently placing the pup in a supine position and observing whether it could right itself and bring all four of its limbs into contact with the surface within 3 s. Those that succeeded were considered to have met the standard. Next, the dates of eye opening (beginning during PNDs 10–16), incisor eruption (beginning on PND 11), and ear opening (beginning on PND 15) were noted. Then, beginning on PND 12, we assessed whether the pups could perform the auditory startle reflex (Ivani et al., 2016), showing a whole-body startle response in response to a loud clap of the hands at less than 15 cm away. These tests were performed until 100% of the pups reached each developmental milestone. In addition, the OFT (PND 21), the Morris Water Maze (MWM) (PND 17–21), and the TST (PND 21) were performed to evaluate the pups’ anxiety, learning, and memory.

The offspring (*n* = 12 per group) were anesthetized with chloral hydrate and sacrificed by decapitation at 21 days after birth. The brain was rapidly removed, and the hippocampus was separated. A proteomic approach using iTRAQ^®^ and a metabolomic approach using LC–MS, ELISA, and western blotting were used to measure changes in proteins in the hippocampus of each offspring to describe the complex molecular mechanisms through which prenatal stress may cause neuronal damage. Another 12 offspring were anesthetized with chloral hydrate, followed by 4% paraformaldehyde perfusion, and their hippocampi were rapidly separated and cut into 1 × 1 mm pieces and immersed in glutaraldehyde to observe the structures of hippocampal neurons through electron microscopy.

#### Open Field Test

OFT behavior was observed following the standard protocol (Smart and Dobbing, 1971), with a few modifications. Briefly, the rats were tested for 5 min in an open-field apparatus that consisted of a bright square with a diameter of 100 cm and a height of 50 cm, with the floor divided into 25 approximately equal sections. The following parameters were observed, using a camera located at a right angle above the open field apparatus (the apparatus was scrubbed with 75% alcohol solution between each test):

Ambulation frequency: number of floor units entered with all four feet
Rearing frequency: number of instances of standing on the hindlimbs without touching the wall
Self-grooming frequency: number of self-grooming actions performed.

#### Morris Water Maze

The Morris Water Maze test was performed following the standard protocol (Wang et al., 2017), with a few modifications. The water maze consisted of a circular pool of water, with a diameter of 100 cm and a height of 60 cm, divided into four equal quadrants by two imaginary perpendicular lines crossing at the center of the pool. Before the experiment, the water maze was filled with tap water to a depth of 45 cm from the brim. The water temperature was controlled at 25–27°C, and it was made opaque by adding potassium permanganate. A stable platform was submerged 1 cm below the surface of the water to allow the rats to easily escape from the water. The training for and administration of the MWM Test had two successive stages: place navigation (initial training) and spatial probe (the Space Exploration Test). During the initial training stage, the rats were trained for 4 days to assess the ability of each rat to obtain spatial information. Each rat was subjected to four trials per day. For each trial, which lasted for 2 min, the platform was placed in the center of the north quadrant. The rat was placed into the water, facing the wall, at one of four starting points (north, south, east, and west) in a semirandom order, and it was allowed to search for the hidden platform. The rat was taken to the cage if it found the platform and stayed on it for about 5 s. However, if the rat did not find the platform within 90 s, it was guided to the platform and allowed to remain there for 30 s. The time spent by the rats to reach the platform (escape latency times) was recorded. After 4 days of training, a spatial probe was administered to evaluate memory retention. The submerged platform was removed from the pool, and the rat was allowed to swim for 90 s in any of the four quadrants of the pool. The number of times that each rat crossed the original platform, the speed of swimming, and the swimming track were recorded with a video-tracking system (CG-400 Image Acquisition System; Institute of Materia Medica, Chinese Academy of Medical Sciences, Shanghai, China). After each trial, the rat was wiped dry and kept warm before being returned to its cage.

#### Sucrose Preference Test

The SPT (Wu et al., 2016) was conducted to investigate the hedonic state of the animals. During the first 2 days of the experiment, the rats were fed separately, and two bottles of 1% sucrose solution were placed in each cage. Then, the rats were fasted from solids and liquids for 12 h. On the third day of the experiment, a bottle containing tap water was substituted for one of the bottles; the other bottle was still filled with 1% sucrose solution. After 1 h, the position of the two bottles was changed to avoid the influence of habitual behavior on the experimental results. After another 1 h, the volume of the remaining 1% sucrose solution and the tap water were measured. The SPT was measured using the following equation: sucrose preference = sucrose consumed / (sucrose consumed + tap water consumed).

#### Tail Suspension Test

The TST was performed following previously described procedures (Wu et al., 2016), with a few modifications. Briefly, the rats were suspended 50 cm above the floor using an adhesive tape placed approximately 4 cm from the tip of the tail for 6 min. Immobility was defined as the absence of movements of the limbs or body, except for those caused by respiration, as the rats hung passively and were completely motionless. During the test, the rats were separated from each other to prevent possible visual and acoustic associations. The results were expressed as the time that the animals spent immobile during the last 4 min of the 6 min session.

#### Equipment for and Method of Preparing the Psychological Fear Stress Model

**FIGURE 1:**
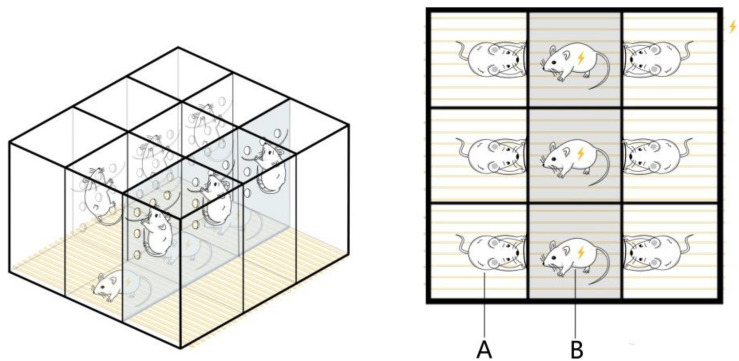
Equipment for the psychological fear stress rat model. **(A)** The pregnant rats can climb onto the wires fixed on the partition to avoid electric shock, but they can observe the pain response behavior of the male and experience fear as a result. **(B)** The male rats cannot avoid electrical stimulation, so they emit painful screams, jump, and lose excretory control during the electrical stimulation.

The pregnant rats were subjected to chronic psychological stress from gestation days 1 to 20, once per day, as described in the following procedure. The electric shock box was modified according to Liping et al. (2013). A 50 × 60 × 60 cm box made of plexiglass plates was divided into nine rooms measuring 20 × 20 × 50 cm, and several holes with a diameter of 1 cm were distributed across the partitions of each room. Iron wires were fixed at the partitions of the six rooms at the edge of the box, and these were used to let the rats climb up and avoid electric shock. The bottom consisted of uniformly arranged stainless steel wires that could be connected to a small electrical stimulator. During the fear stress experiment, three male rats were put into the three middle rooms for electrical stimulation treatment, with one rat in each room. The pregnant rats from the FSM group were put into the six rooms along the edge, with one rat in each room. After the power was switched on, the male rats were shocked, causing them to scream, jump, and lose excretory control. While the pregnant rats from the model group could climb onto the wires fixed on the partition to avoid electric shock, they could still perceive the males being shocked through hearing, vision, and smell, causing fear (Figure 1). During the stress stimulation, a stopwatch was set for 60 s, and after the timer was finished, the alarm bell rang continuously for 60 s. During the bell ringing, the male rats were stimulated 10 times with instantaneous 25–35 V, which was repeated 15 times. The pregnant rats from the NC group were also placed in the electric shock box for 30 min every day, but no electrical stimulation was performed, and all other operations were the same as those in the model group.

### iTRAQ-Based Proteomics Analyses

#### Protein Extraction

A sample of the hippocampus of each animal was added to liquid nitrogen to be ground into powder and mixed well with 6× volume of chilled trichloroacetic acid (TCA)–acetone (500 ml acetone, 50 g TCA) and incubated at −20°C for at least 2 h. After centrifugation at 4°C and 20,000 × *g* for 30 min, the supernatant was discarded, and the precipitate was dissolved with lysis buffer (8 M urea, 30 mM 4-(2-hydroxyethyl)-1-piperazineethanesulfonic acid, 1 mM phenylmethylsulfonyl fluoride, 2 mM ethylenediaminetetraacetic acid, and 10 mM dithiothreitol) and sonicated at 180 W 30 times (pulse on for 2 s and pulse off for 3 s for each time). After being centrifuged at 4°C and 20,000 × *g* for 30 min, the supernatant was collected and incubated at 56°C for 1 h, then added to iodoacetamide (55 mM), and incubated in the dark for 1 h and subsequently mixed well with 4 × volume of chilled TCA–acetone before being incubated at −20°C for at least 3 h. After centrifugation at 4°C and 20,000 × *g* for 20 min, the supernatant was discarded, and the precipitate was redissolved in 300 μl dissolution buffer [50% tetraethylammonium bromide (TEAB) and 0.1% sodium dodecyl sulfate (SDS)] and sonicated at 180 W for 3 min. The protein concentrations were quantified using the Bradford method [Pierce Coomassie (Bradford) Protein Assay Kit, Thermo Fisher Fisher].

#### Protein Processing and iTRAQ Labeling

Total protein (100 μg) from each sample was added to the dissolution buffer (50% TEAB and 0.1% SDS). Then, 3.3 μg trypsin was added to each sample, and the samples were incubated at 37°C for 24 h. This was repeated with 1 μg trypsin incubated at 37°C for 12 h to digest the protein. Subsequently, the peptide segment was freeze-dried and redissolved with 30 μl dissolution buffer (50% TEAB and 0.1% SDS). The digestion efficiency was quantified *via* MALDI Tof/Tof. The sample was labeled with iTRAQ reagent, as follows. The tryptic peptides were labeled with an iTRAQReagent-8Plex Multiplex Kit (Applied Biosystems), following the manufacturer’s protocol. The NC group was labeled with iTRAQ reagent 119, and the FSM group was labeled with iTRAQ reagent 121. Incubation was allowed to proceed at room temperature for 2 h and then was stopped by adding 10 mM KH_2_PO_4_ and 25% acetonitrile (ACN) at pH = 3.0. Subsequently, the two labeled samples were pooled and preliminarily separated using strong cation exchange (SCX) high-performance liquid chromatography. Briefly, the peptides were dissolved and loaded onto an SCX chromatographic column (Luna SCX 250 × 4.60 mm 100 Å, phenomenex) in buffer A (10 mM KH_2_PO_4_ in 25% ACN, pH = 3.0) and equilibrated in 100% solution A for 10 min, followed by fast elution in a gradient of 0–10% B (2 M KCL and 10 mM KH_2_PO_4_ in 25% ACN, pH = 3.0, flow rate = 1 ml/min) for 5 min, 10–20% B for 20 min, 20–30% B for 5 min, and 30–50% solution B for 3 min. The fractioned peptides were collected at a rate of one tube per minute during the elution period. Each fraction was desalted using a C18 column (strata-X C18, phenomenex), lyophilized, and dissolved in 0.1% formic acid for LC–MS/MS analyses (Chen et al., 2016).

### LC–MS/MS Analyses

The peptides in each fraction were delivered into EASY-nLC (PROXEON) and eluted with 5–45% ACN in 0.1% formic acid for 90 min at 300 nl/min. The peptides eluted from LC were analyzed using a hybrid quadrupole/time-of-flight mass spectrometer (microTOF-Q II, Bruker) (Chen et al., 2016). The source parameters were as follows: capillary voltage, 1,350 V; dry gas, 3.0 L/min; and dry temperature, 150°C. The acquisition range was 300–1,500 m/z for the MS and 50–3,000 m/z for the MS/MS. The absolute threshold for both MS and MS/MS was 1,000 intensity counts. The spectra time for MS was set to 0.5 s, that for MS/MS with 1,000–10,000 intensity counts was set to 1.2 s, and that for MS/MS with >10,000 intensity counts was set to 0.5 s. The auto MS/MS mode was used with positive ion polarity, and the preferred range of the charge state was set from 2+ to 5+, with no single charge. The collision energy for fragmentation was set to certain dynamic ranges for different charge states: 12–72 eV for 1+, 22–62 eV for 2+, 17–57 eV for 3+, 15–55 eV for 4+, and 12–52 eV for 5+. Collision energy sweeping was set as follows: start 100% (timing 55%) and end 100% (timing 45%). Three precursor ions were collected per cycle with active exclusion (1 min).

### Data Analyses

Raw data files were converted into MGF files using Proteome Discoverer 1.3 (Thermo Fisher Scientific). The proteins were identified using Mascot 2.3.0 (Matrix Science, Boston, MA, United States), based on the ipi-rat-v3-85-1 database and quantified using Proteome Discoverer 1.3. The user-defined search parameters were as follows: fixed modification to carbamidomethyl (C), variable modification to oxidation (M), Gln-Pyro-Glu to N-term Q, iTRAQ 8-plex to K, iTRAQ 8-plex to Y, iTRAQ 8-plex to N-term, enzyme to trypsin, peptide tolerance to 0.1 Da, MS/MS tolerance to 0.1 Da, and maximum missed cleavages to 1. The quantitative analysis parameters were set as follows: protein ratio type to median, minimum peptides to 1, peptide threshold type to at least homology, normalization method to median, *P*-value to < 0.05, peptide false discovery rate to < 1%, and iTRAQ reporter ratio change to > 1.2-fold or < 0.83-fold.

### Bioinformatics Analysis

The STRING database and Cytoscape were used for the construction of protein–protein interaction networks. GO analyses were performed to analyze the main functions of the DEPs, and KEGG (Kanehisa Laboratories, Kyoto, Japan) was used to identify the significant pathways of the DEPs. The Cytoscape ClueGO software tool was used to perform GO analyses and pathway analyses (Bindea et al., 2009; Liu et al., 2019; Xu et al., 2019). The parameters of the analyses were as follows: hyper-geometric distribution, two-sided (enrichment/depletion) tests, statistical significance of *p* ≤ 0.05, correction with the Bonferroni adjustment, a Kappa-statistic score of 0.4, GO-level intervals of 3–8, leading group at highest significance, and group merge of 50%.

### Western Blot

The hippocampus samples were lysed in RIPA Lysis Buffer (Beyotime Biotechnology, Shanghai, China, catalog P0013B). The lysates were incubated on ice for 10 min and centrifuged at 4°C and 12,000 × *g* for 5 min, and then the supernatants were collected. The protein concentrations were quantified using a bicinchoninic acid kit (Tiangen, China). The protein lysates were separated by SDS- polyacrylamide gel electrophoresis (4–6%) and transferred to a polyvinylidene fluoride membrane (Millipore, Schwalbach, Germany). After blocking with 5% skimmed milk in Tris-buffered saline Tween-20 at room temperature for 1 h, the membranes were incubated with the following primary antibodies against PKG (CST, catalog 3248, Boston, MA, United States) in Tris-buffered saline Tween-20 at 4°C overnight. After washing, the membranes were incubated in horseradish peroxidase-conjugated secondary antibody (1:1,000) at room temperature for 1 h. The bound antibodies on the membranes were visualized using enhanced chemiluminescence. With glyceraldehyde 3-phosphate dehydrogenase, the antibody was used as a control for protein loading. The relative level of each protein to the control protein was quantified using Image-Pro Plus 6.0 software (Media Cybernetics, Rockville, MD, United States).

### Electron Microscopy

Transmission electron microscope images were prepared by the Electron Microscope Center of Scientific Research and Experiment Center of Henan University of Chinese Medicine. Tissue pieces of the hippocampus were fixed with 2.5% glutaraldehyde for 4 h and washed four times with 0.1 M phosphate-buffered saline (PBS) for 15 min each time. After rinsing, the samples were placed into 1% osmium acid fixative solution and fixed again for 1.5 h. Then, the samples were rinsed with 0.1 M PBS for 15 min each time. The tissues were dehydrated with graded alcohol series (50, 70, 80, and 100%) and embedded in a mixture of epoxy resin 812 and acetone (1:1), epoxy resin 812 and acetone (2:1), and pure epoxy resin 812, respectively, and placed overnight at room temperature. Then, these were sliced into serial coronal 50–60 nm thick sections using an ultra-thin slicing machine and dyed in saturated uranium dioxide acetate solution for 20 min. After rinsing and drying, the sections were observed and photographed by transmission electron microscopy.

### Morphometric Analysis of Mitochondria

Morphometric analyses of mitochondria were performed with the Fiji Software on a sample of 10 systematically, uniformly, and randomly selected images (Paukov et al., 1971). First, the mean number of mitochondria per electron micrograph was calculated. The second index of the state of the mitochondria ultrastructure was the mean number of cristae per mitochondrion. The third index of the state of the mitochondria ultrastructure was the area of the mitochondria, directly reflecting the volume of these organelles. The fourth index of the state of the mitochondria ultrastructure was the coefficient of energy efficiency of mitochondria (CEEM). This coefficient is the product of the number of mitochondrial cristae and the area of mitochondria.

### Statistical Analyses

Measurement data are presented as means ± SDs, and the statistical analyses were performed using SPSS 21.0 statistical software (IBM, Armonk, NY, United States). The scores for the SPT, TST, and OFT for pregnant rats and the escape latency of offspring were analyzed using repeated-measures ANOVA. An independent *t*-test was conducted to analyze the body weights of the pregnant rats and the birth weights and scores of the offspring. Two-way ANOVA was conducted to analyze the TST, OFT, and Space Exploration Test of the offspring. *P* < 0.05 was considered as statistically significant.

## Results

### Weight Changes in Pregnant Rats During Gestation

**FIGURE 2:**
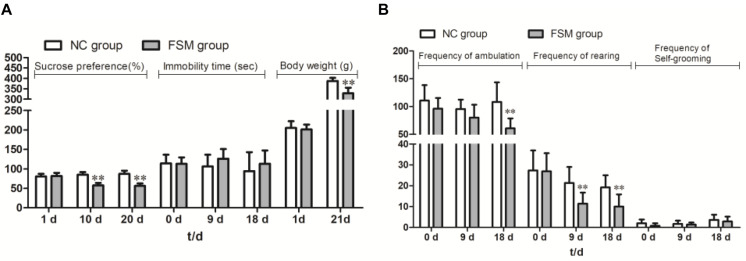
Behavioral assessment of pregnant rats. The pregnant rats’ results for **(A)** the Sucrose Preference Test, immobility time, and body weight changes and **(B)** the Open Field Test are presented. Values are expressed as means ± standard deviations. NC group, normal control group (*n* = 10); FSM group, fear stress model group (*n* = 12). **P < 0.01 versus the NC group.

Because some females were not pregnant after the mating period, five rats were excluded from the NC group and three from the FSM group, leaving 10 rats in the NC group and 12 rats in the FSM group. As shown in Figure 2A, the dams in the NC and FSM groups had similar body weights before exposure to stress. However, after treatment, the females in the FSM group had lower body weights. On day 21 of pregnancy, their body weights were significantly less than those of the NC group (*t* = 6.251, *P* < 0.01).

### Behavioral Assessment of Pregnant Rats

Repeated-measures ANOVA showed that stimulation time had a significant effect on sucrose consumption (*F* = 12.535, *P* < 0.001). An interaction was found between stimulation time and group (*F* = 32.433, *P* < 0.001), and the sucrose consumption of the FSM rats was significantly less than that of the NC rats (*P* < 0.001) (Figure 2A). For the TST results (Figure 2A), a repeated-measures ANOVA showed that there were no differences between the groups (*P* > 0.05) and no effects of stimulation time (*F* = 1.264, *P* > 0.05). In addition, for the OFT results (Figure 2B), stimulation time had a significant effect on the frequency of ambulation (*F* = 5.684, *P* < 0.01), rearing (*F* = 19.538, *P* < 0.001), and self-grooming (*F* = 7.123, *P* < 0.01). There were significant differences between the groups in terms of ambulation (*P* < 0.01) and rearing (*P* < 0.01). These results suggest that the exploratory behavior of rats in the FSM group was decreased.

### Physical and Reflex Development of the Offspring Rats

**FIGURE 3:**
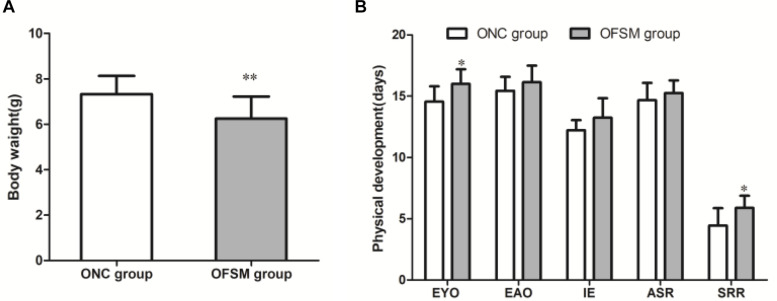
Fear stress in pregnant rats during gestational days may affect the physical and the reflex development of offspring. **(A)** Birth weights of offspring. ONC group (*n* = 125) and OFSM group (*n* = 129). **(B)** Physical and reflex development of offspring. OFSM, offspring from the FSM group (*n* = 12); ONC, offspring from the normal control group (*n* = 10); EYO, eye opening; EAO, ear opening; IE, incisor eruption; ASR, auditory startle reflex; SRR, surface righting reflex. Values are expressed as means ± standard deviations. *P < 0.05 and **P < 0.01 versus the ONC group.

The birth weights of the offspring of the FSM group (OFSM, 7.33 ± 0.80) were significantly lower than those of the offspring of the NC group (ONC, 6.25 ± 0.97, *t* = 9.628, *P* < 0.01, Figure 3A). This suggests that fear stress in pregnant rats affected the birth weights of their offspring. Among indicators of the physical and the reflex development of the offspring, the eye opening (*t* = *-*2.720, *P* < 0.05) and the surface righting reflex (*t* = *-*2.694, *P* < 0.05, Figure 3B) times of the pups in the OFSM group were significantly delayed compared to those of the ONC group. No significant differences were observed in ear opening, incisor eruption, or auditory startle reflex between the two groups (*P* > 0.05, Figure 3B). These results suggest that fear stress may have also led to a delay in the physiological and the reflex development of offspring.

### Behavioral Assessment of Offspring

**FIGURE 4:**
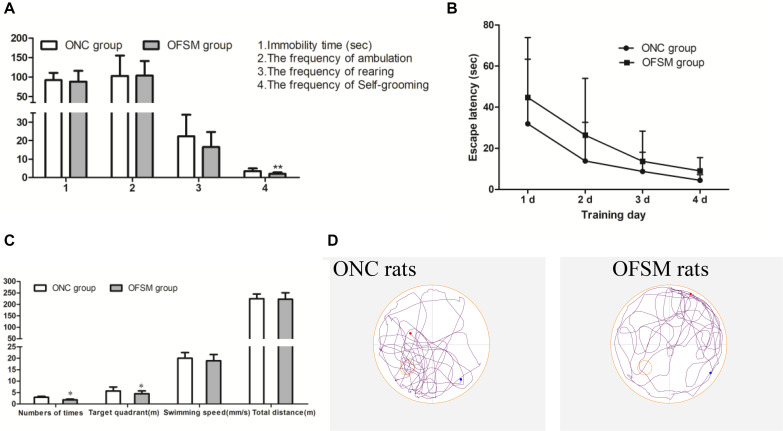
Fear stress in pregnant rats may affect the behavior of their offspring. **(A)** Immobility times in the TST and the frequency of ambulation, rearing, and self-grooming in the OFT. **(B)** Escape latency during the four training days in the MWM. **(C)** Swimming speeds and distances during the probe test in the MWM. **(D)** Representative MWM-generated swimming tracing pattern for offspring from the normal control group (ONC) and for offspring from the fear stress model (OFSM) group in the probe tests. TST, Tail Suspension Test; OFT, Open Field Test; MWM, Morris Water Maze. Values are expressed as means ± standard deviations. ONC group (*n* = 19), OFSM group (*n* = 18). *P < 0.05, **P < 0.01 versus ONC group.

To observe the psychological status of offspring, behavioral tests, including the TST, OFT, and MWM Test, were conducted on PND 21. In the TST, there were no significant differences in immobility time between the two offspring groups (*F* = 0.445, *P* > 0.05, Figure 4A). In addition, in the OFT scores (Figure 4A), there were no significant differences in the frequency of ambulation (*F* = 0.003, *P* > 0.05) or frequency of rearing (*F* = 1.601, *P* > 0.05) between the two offspring groups. However, the frequency of self-grooming was significantly lower in OFSM rats (*F* = 9.924, *P* < 0.01, Figure 4D), and there was no significant difference between different genders (*P* > 0.05). Learning occurred in both the ONC and the OFSM groups as the escape latencies became progressively shorter, but the escape latencies for the OFSM rats were longer than those of ONC rats. A repeated-measures ANOVA showed that the differences between different groups (*F* = 3.55, *P* = 0.068, Figure 4B) and genders (*F* = 0.09, *P* = 0.766) were not statistically significant. The results of the probe test for OFSM are shown in Figure 4C. There were no significant differences in swimming speed (*F* = 0.105, *P* > 0.05, Figure 4C) and swimming distance (*F* = 1.665, *P* > 0.05, Figure 4C) between the two groups. The swimming distance in the target quadrant (*F* = 6.03, *P* < 0.05) and the number of times that the rats crossed the original platform (*F* = 4.413, *P* < 0.05) of the OFSM group significantly decreased relative to the values for the ONC rats (Figure 4C). There was no significant difference between different genders (*P* > 0.05). Overall, these results indicate that fear stress in pregnant rats might impair the spatial learning and the memory ability of their offspring. Representative tracks of the probe test are shown in Figure 4D.

### iTRAQ-Based Proteomic Analyses

Next, we investigated the effects of chronic fear stress in pregnant rats on the expression of proteins in the hippocampi of their pups using iTRAQ-based proteomics analyses. Matching to the ipi-rat-v3-85-1 database, within the two groups, a total of 987 proteins that contained at least one unique peptide were identified, and 0.2% false discovery rate was used for multivariate analyses. iTRAQ reporter ratios that changed > 1.2-fold or < 0.83-fold and *P* < 0.05 versus the values for the NC group were deemed to be DEPs. Overall, 158 DEPs were found between the two groups. Of these, 58 proteins were upregulated (Table 1) and 100 were downregulated (Table 2) in the OFSM group, relative to the ONC group, that is, the number of downregulated proteins was much higher than the number of upregulated proteins.

**TABLE 1:** Upregulated proteins in the hippocampus of pups.

Accession	Protein names
D3ZJK2	Serine (or cysteine) peptidase inhibitor, clade B (ovalbumin), member 3A (Serpinb3a)
Q04940	Neurogranin (Nrgn)
P97546	Neuroplastin (Nptn)
D3ZTW5	Solute carrier family 25, member 54 (Slc25a54)
P34926	Microtubule-associated protein 1A (Map1a)
Q6IMY8	Heterogeneous nuclear ribonucleoprotein U (Hnrnpu)
P08461	Dihydrolipoamide S-acetyltransferase (Dlat)
Q6P799	Seryl-tRNA synthetase (Sars)
P09456	Protein kinase cAMP-dependent type 1 regulatory subunit alpha (Prkar1a)
D3ZXP3	H2A histone family, member X (H2afx)
O35987	NSFL1 cofactor (Nsfl1c)
P60711	Actin, beta (Actb) (fragment)
O88201	C-type lectin domain family 11, member A (Clec11a)
O35331	Pyridoxal (pyridoxine, vitamin B6) kinase (Pdxk)
Q6P6R2	Dihydrolipoamide dehydrogenase (Dld)
Q68FP8	Adenylate kinase 8 (Ak8)
Q5FVQ4	Malectin (Mlec)
M0R5M3	RGD1562660 (RGD1562660)
Q62658	FK506 binding protein 1a (Fkbp1a)
Q6PDU1	Serine/arginine-rich splicing factor 2 (Srsf2)
P27139	Carbonic anhydrase 2 (Car2)
P04550	Parathymosin (Ptms)
Q56R17	Karyopherin subunit alpha 4 (Kpna4)
P0C5X8	Tweety family member 1 (Ttyh1)
Q7TNA8	Lactate dehydrogenase A-like 6B (Ldhal6b)
P52296	Karyopherin subunit beta 1 (Kpnb1)
F1M4G6	ENSRNOG00000023547 uncharacterized protein (fragment)
Q05546	Tenascin R (Tnr)
B0BNB2	Density-regulated re-initiation and release factor (Denr)
Q6P7Q4	Glyoxalase 1 (Glo1)
Q6AY09	Heterogeneous nuclear ribonucleoprotein H2 (H’) (Hnrnph2)
P62959	Histidine triad nucleotide binding protein 1 (Hint1)
NP_112406	Actin, beta (Actb) (fragment)
Q9Z0V6	Peroxiredoxin 3 (Prdx3)
P47987	Plasmolipin (Pllp)
Q6P9T8	Tubulin, beta 4B class IVb (Tubb4b)
P28480	T-complex 1 (Tcp1)
Q3MHS9	Chaperonin containing TCP1 subunit 6A (Cct6a)
Q9JHL4	Drebrin-like (Dbnl)
O88767	Parkinsonism-associated deglycase (Park7)
Q4V7A0	WD repeat domain 61 (Wdr61)
O08589	FXYD domain-containing ion transport regulator 1 (Fxyd1)
Q9EPR9	Phosphodiesterase 1A (Pde1a)
Q3ZBA0	Tectonin beta-propeller repeat containing 1 (Tecpr1)
R9PXY3	Phospholipase C beta 1 (Plcb1)
Q5XIM7	Lysyl-tRNA synthetase (Kars)
P85972	Vinculin (Vcl)
P04797	Glyceraldehyde-3-phosphate dehydrogenase (Gapdh)
M0R5U4	Adenylate cyclase 9 (Adcy9)
P02688	Myelin basic protein (Mbp)
A0A0G2JSZ5	Protein disulfide isomerase family A, member 6 (Pdia6)
Q32PX7	Far upstream element binding protein 1 (Fubp1)
Q2QC85	Far upstream element binding protein 3 (Fubp3)
Q5U2Z3	Nucleosome assembly protein 1-like 4 (Nap1l4)
Q9Z2L0	Voltage-dependent anion channel 1 (Vdac1)
Q63014	A-kinase anchoring protein 8 (Akap8)
Q03346	Peptidase, mitochondrial processing beta subunit (Pmpcb)
Q5I0I7	Similar to T cell receptor V-alpha J-alpha (LOC290071)

**TABLE 2:** Downregulated proteins in the hippocampus of pups.

Accession	Protein names
Q9Z270	VAMP-associated protein A (Vapa)
Q6PCU0	ATP synthase, H+ transporting, mitochondrial F1 complex, gamma polypeptide 1 (Atp5c1)
Q63356	Myosin IE (Myo1e)
Q5XI32	Capping actin protein of muscle Z-line beta subunit (Capzb)
P10960	Prosaposin (Psap)
NP_001164074	Astrotactin 1 (Astn1)
D3ZMR1	Translocase of outer mitochondrial membrane 7 (Tomm7)
F1LSW6	Diphosphoinositol pentakisphosphate kinase 1 (Ppip5k1)
P21670	Proteasome subunit alpha 4 (Psma4)
P37996	ADP ribosylation factor-like GTPase 3 (Arl3)
A0A0G2K2B5	Aspartate-beta-hydroxylase (Asph)
P09495	Tropomyosin 4 (Tpm4)
D4ACN7	Myoferlin (Myof)
P63170	Dynein light chain LC8-type 1 (Dynll1)
F1M8X9	Golgi brefeldin A resistant guanine nucleotide exchange factor 1 (Gbf1)
Q5XIC6	Proteasome 26S subunit, non-ATPase 12 (Psmd12)
Q68FS4	Leucine aminopeptidase 3 (Lap3)
Q6AYL4	RIB43A domain with coiled-coils 1 (Ribc1)
Q2TGJ4	Zinc finger, DHHC-type containing 15 (Zdhhc15)
P12369	Protein kinase cAMP-dependent type 2 regulatory subunit beta (Prkar2b)
Q9Z1P2	Actinin, alpha 1 (Actn1)
P19511	ATP synthase, H+ transporting, mitochondrial Fo complex, subunit B1 (Atp5f1)
P62762	Visinin-like 1 (Vsnl1)
P63100	Protein phosphatase 3, regulatory subunit B, alpha (Ppp3r1)
P68511	Tyrosine 3-monooxygenase/tryptophan 5-monooxygenase activation protein, eta (Ywhah)
P50408	ATPase H+ transporting V1 subunit F (Atp6v1f)
D4ACB8	Chaperonin containing TCP1 subunit 8 (Cct8)
P62260	Tyrosine 3-monooxygenase/tryptophan 5-monooxygenase activation protein, epsilon (Ywhae)
P70566	Tropomodulin 2 (Tmod2)
Q5I0D5	Phospholysine phosphohistidine inorganic pyrophosphate phosphatase (Lhpp)
Q01986	Mitogen-activated protein kinase 1 (Map2k1)
P04631	S100 calcium binding protein B (S100b)
D3ZLT1	NADH:ubiquinone oxidoreductase subunit B7 (Ndufb7)
P32736	Opioid binding protein/cell adhesion molecule-like (Opcml)
O70257	Syntaxin 7 (Stx7)
Q08163	Adenylate cyclase associated protein 1 (Cap1)
P61983	Tyrosine 3-monooxygenase/tryptophan 5-monooxygenase activation protein, gamma (Ywhag)
Q5XIL1	ATPase H+ transporting V1 subunit H (Atp6v1h)
P63182	Cerebellin 1 precursor (Cbln1)
P17220	Proteasome subunit alpha 2 (Psma2)
Q5MJ12	F-box and leucine-rich repeat protein 16 (Fbxl16)
P31399	ATP synthase, H+ transporting, mitochondrial Fo complex, subunit d (Atp5h)
Q925N3	Peroxisomal biogenesis factor 5-like (Pex5l)
D4ADD7	Glutaredoxin 5 (Glrx5)
Q63617	Hypoxia up-regulated 1 (Hyou1)
P62882	G protein subunit beta 5 (Gnb5)
O35964	SH3 domain-containing GRB2-like 1 (Sh3gl1)
P07171	Calbindin 1 (Calb1)
P60881	Synaptosomal-associated protein 25 (Snap25)
Q6PCU2	ATPase H+ transporting V1 subunit E1 (Atp6v1e1)
Q8VBU2	NDRG family member 2 (Ndrg2)
D3ZLI4	IQ domain-containing protein F5-like (LOC102550160)
P47858	Phosphofructokinase, muscle (Pfkm)
D3ZAZ5	Breast cancer anti-estrogen resistance 3 (Bcar3)
Q64119	Myosin, light polypeptide 6, alkali, smooth muscle and non-muscle-like (Myl6l)
AAH91292	Pyruvate dehydrogenase complex, component X (Pdhx)
B0K020	CDGSH iron sulfur domain 1 (Cisd1)
A0A096MJI5	Ligase III, DNA, ATP-dependent (Lig3)
P30904	Macrophage migration inhibitory factor (glycosylation-inhibiting factor) (Mif)
Q63347	Proteasome 26S subunit, ATPase 2 (Psmc2)
G3V8D5	6-Phosphogluconolactonase (Pgls)
D3ZCV0	Actinin alpha 2 (Actn2)
P62628	Dynein light chain roadblock-type 1 (Dynlrb1)
Q62760	Translocase of outer mitochondrial membrane 20 (Tomm20)
P84076	Hippocalcin (Hpca)
Q5XI34	Protein phosphatase 2 scaffold subunit A alpha (Ppp2r1a)
Q68FS0	Similar to 14-3-3 protein sigma (LOC298795)
A0A0H2UHP9	RAB6A, member RAS oncogene family (Rab6a)
P05197	Eukaryotic translation elongation factor 2 (Eef2)
P37397	Calponin 3 (Cnn3)
Q05962	Solute carrier family 25 member 4 (Slc25a4)
D3ZLI9	PDZ domain containing 4 (Pdzd4)
Q6AYD3	Proliferation-associated 2G4 (Pa2g4)
Q9Z272	GIT ArfGAP 1 (Git1) (fragment)
D4AAD6	Neuropilin and tolloid-like 1 (Neto1)
Q66HF1	NADH dehydrogenase (ubiquinone) Fe–S protein 1 (Ndufs1)
D3ZVQ0	Ubiquitin-specific peptidase 5 (Usp5)
D4AEF2	Olfactory receptor 813 (Olr813)
P63055	Purkinje cell protein 4 (Pcp4)
P35213	Tyrosine 3-monooxygenase/tryptophan 5-monooxygenase activation protein, beta (Ywhab)
P85973	Purine nucleoside phosphorylase (Pnp)
A1L1J8	RAB5B, member RAS oncogene family (Rab5b)
Q63560	Microtubule-associated protein 6 (Map6)
Q9WU82	Catenin beta 1 (Ctnnb1)
P47863	Aquaporin 4 (Aqp4)
P19944	Ribosomal protein, large, P1 (Rplp1)
F7F2F3	Heat shock protein 4-like (Hspa4l)
P68255	Tyrosine 3-monooxygenase/tryptophan 5-monooxygenase activation protein, theta (Ywhaq)
P04904	Glutathione S-transferase alpha 1 (Gsta1) (fragment)
D3ZC55	Heat shock protein family A (Hsp70) member 12A (Hspa12a)
P48768	Solute carrier family 8 member A2 (Slc8a2)
P43527	Caspase 1 (Casp1)
Q6Q0N1	CNDP dipeptidase 2 (metallopeptidase M20 family) (Cndp2)
F1LPG5	NADH:ubiquinone oxidoreductase subunit B4 (Ndufb4)
Q5FVI6	ATPase H+ transporting V1 subunit C1 (Atp6v1c1)
D3Z8Q5	Tumor suppressor candidate 2 (Tusc2)
P30835	Phosphofructokinase, liver type (Pfkl)
Q4KLN7	ADP-ribosylation factor GTPase activating protein 3 (Arfgap3)
P08592	Amyloid beta precursor protein (App) (fragment)
Q62703	Reticulocalbin 2 (Rcn2)

### GO and KEGG Enrichment Analyses and Protein–Protein Interaction Network of DEPs

**FIGURE 5:**
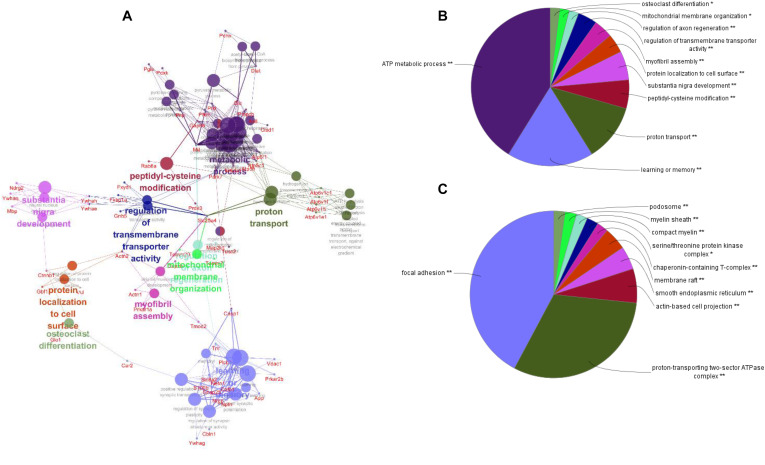
The network of biological processes for the differentially expressed proteins (DEPs) **(A)**. The biological processes [Gene Ontology (GO) category] of the DEPs were visualized using ClueGO software (*p* ≤ 0.05, kappa≥ 0.4), and only the most significant interactions are shown. Each node represents a biological process, and the size of each node reflects the enrichment significance of the GO terms. The edges represent connections between the nodes. The color of the node represents the class. Mixed coloring represents multiple classes. Ungrouped terms are not shown. The distribution of DEPs according to biological process **(B)** and cellular location **(C)** was determined by ClueGO (*p* ≤ 0.05, kappa ≥ 0.4), and only the most significant terms are shown. *P < 0.05, **P < 0.01.

**FIGURE 6:**
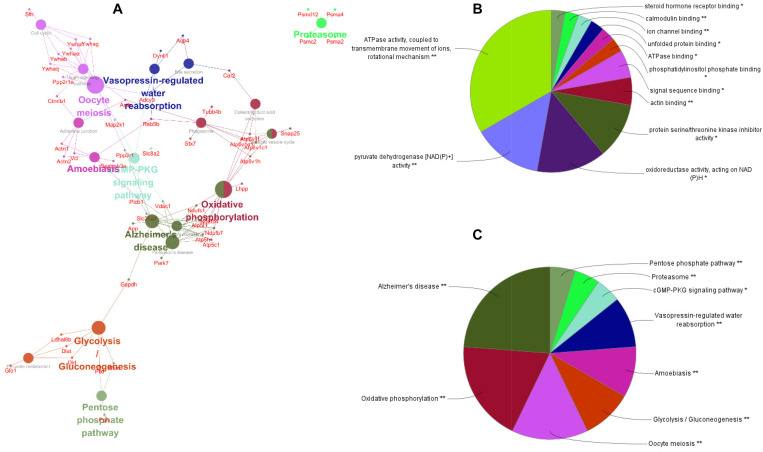
The pathway network of the differentially expressed proteins **(A)** visualized using ClueGO software (*p* ≤ 0.05, kappa ≥ 0.4); only the most significant interactions are shown. Network nodes represent pathways, and the sizes of the nodes reflect the enrichment significance of the GO terms. Edges represent connections between nodes. The color of the node represents the class it belongs to. Mixed coloring represents multiple classes. Ungrouped terms are not shown. Molecular function **(B)** and pathway enrichment **(C)** are determined by ClueGO (*p* ≤ 0.05, kappa ≥ 0.4), and only the most significant terms are shown. *P < 0.05, **P < 0.01.

**FIGURE 7:**
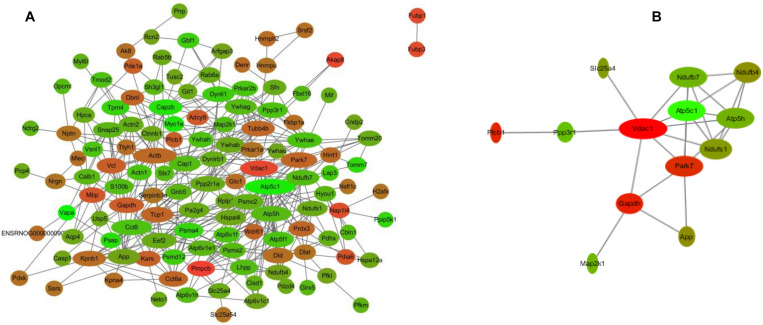
The interaction network of differentially expressed proteins (DEPs) **(A)** and the interaction network of DEPs related to learning or memory and cyclic guanosine monophosphate–PKG pathway **(B)**. Network nodes represent proteins; edges represent protein–protein associations. Red, significantly increased; green, significantly decreased; width, degree.

To better understand the mechanisms of the effects of mental stress in pregnant rats on the learning and memory of their offspring, GO classification and pathway enrichment of the DEPs were performed. The results suggested that fear stress during pregnancy affected offspring metabolism, biosynthetic function, transport function, learning, and memory, among other impacts. According to GO classification, the DEPs were involved in osteoclast differentiation (30316), mitochondrial membrane organization (7006), regulation of axon regeneration (48679), regulation of transmembrane transporter activity (22898), myofibril assembly (30239), protein localization to cell surface (34394), substantia nigra development (21762), peptidyl-cysteine modification (18198), proton transport (15992), learning or memory (7611), ATP metabolism (46034), and other biological processes (Figure 5A). The DEPs were mainly enriched in the ATP metabolic process and learning or memory (Figure 5B). According to the GO cellular component annotations, the DEPs were mainly located in focal adhesion (0005925), proton-transporting two-sector ATPase complex (16469), and actin-based cell projection (98858) (Figure 5C). The GO molecular function annotations showed that the DEPs were involved in ATPase activity, coupled to the transmembrane movement of ions, rotational mechanisms (0044769), pyruvate dehydrogenase [NAD(P)+] activity (0034603), oxidoreductase activity, acting on NAD(P)H (0016651), and protein serine/threonine kinase inhibitor activity (0030291) (Figure 6B). In pathway enrichment analyses, the DEPs were related to the pentose phosphate pathway (00030), proteasome (03050), the cyclic guanosine monophosphate-protein kinase G (cGMP–PKG) signaling pathway (04022), vasopressin-regulated water reabsorption (04962), amoebiasis (05146), glycolysis/gluconeogenesis (00010), hippo signaling pathway (04390), oxidative phosphorylation (00190), and Alzheimer’s disease (05010) (Figure 6A). According to pathway enrichment analyses, the DEPs were mainly enriched in Alzheimer’s disease, oxidative phosphorylation, oocyte meiosis (04114, or the hippo signaling pathway, 04390), and the glycolysis/gluconeogenesis pathways (00010) (Figure 6C). The protein–protein interaction network of these proteins is shown in Figure 7. Analyses of this network revealed that ATP5C1, VDAC1, ACTB, GAPDH, and PARK7 participated in a range of biological processes. ATP5C1 interacts with multiple proteins (ATP5C1, ATP5H, ATP5F1, DLD, ATP6V1F, ATP6V1E1, and NDUFS1) that participate in oxidative phosphorylation and the ATP metabolic process. VDAC1 interacts with YWHAE, APP, PPP3R1, YWHAB, CASP1, VDAC1, SFN, NDUFS1, DYNLL1, and GAPDH, which participate in cell death. PARK7 interacts with multiple proteins, which participate in behavior, oxygen and reactive oxygen species metabolism, regulation of neurotransmitter levels, and synaptic transmission. These changes may play an important role in the impairment of learning and memory function in offspring.

### Effects of Chronic Fear Stress in Pregnant Rats on the cGMP/PKG Pathway in the Hippocampus of Offspring

**FIGURE 8:**
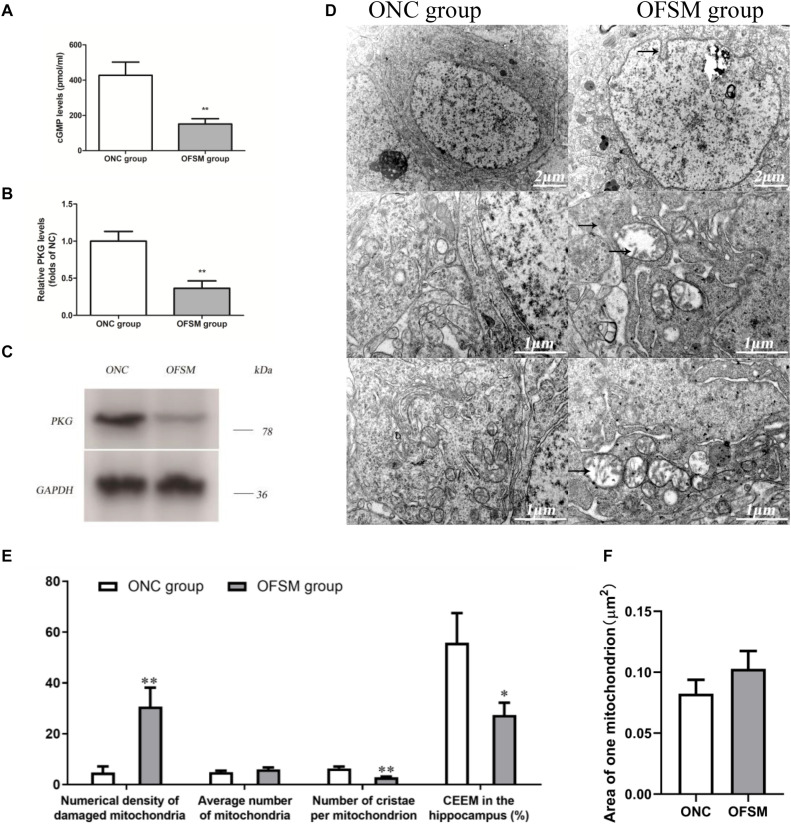
The levels of cyclic guanosine monophosphate (cGMP) and protein kinase G (PKG) expression in the hippocampus of rats were detected by ELISA and western blotting assay, and the ultrastructure of the neurons was observed by electron microscopy. **(A)** Levels of cGMP. **(B)** PKG protein expression determined by western blotting. **(C)** Representative western blotting image of PKG; the results are presented as means ± SD (*n* = 6). **P < 0.01 versus offspring from the normal control group. **(D)** Representative electron microscopy pictures of hippocampal neurons (the ultrastructural changes are indicated by a black arrow; *n* = 4 for each group). **(E)** Morphometric analysis of structural changes in the mitochondria of the hippocampus; the results are presented as means ± SEM. Magnification × 50,000. **(F)** The percent numerical density of damaged mitochondria in the hippocampus; the results are presented as means ± SEM. Magnification ×50,000. *P < 0.05, **P < 0.01 versus offspring from the normal control group.

Pathway enrichment analyses show that a rather high number of DEPs are related to the Alzheimer’s disease pathway. As shown in Figure 6A, a disturbance in the cGMP–PKG signaling pathway of the hippocampus was involved in learning and memory impairment. Thus, the cGMP level and the expression of PKG protein in the hippocampus were further analyzed using ELISA and western blotting. The results are shown in Figure 8. We found that the cGMP level and the PKG protein expression were significantly lower in the hippocampus of rats in the OFSM group than in those of the ONC group (*P* < 0.01). These results suggest that prenatal stress inhibited the cGMP/PKG signaling pathway, which led to memory impairment in postnatal rats.

### Effects of Chronic Fear Stress in Pregnant Rats on the Ultrastructure of Neurons in the Hippocampus of Offspring

As shown in Figure 6, according to the pathway enrichment and network analyses, high numbers of DEPs were related to oxidative phosphorylation, which is involved in learning and memory impairment. Oxidative phosphorylation in the mitochondria is the main site for the production of ATP in neuronal cells, so the ultrastructure of the neurons in the hippocampus of the offspring was observed with electron microscopy. The results are shown in Figure 8D. The neuronal shape, nuclear membrane, and nucleolus were clearly visible in the ONC group, chromatin was distributed relatively evenly, the organelle structure was normal, the mitochondrial membrane and the mitochondrial cristae were distinct, the complete outline of the synaptic membrane and the synaptic cleft was clear, and a large amount of bright round vesicles was seen distributed in the presynaptic terminals, while the postsynaptic density was thick or long in the hippocampal region. In the OFSM group, the synaptic structure was abnormal, the membranes of pre-axoaxonic synapses and post-axoaxonic synapses were not clear, the synaptic space disappeared, and the number of synapse vesicle was decreased. The neuronal mitochondria appeared swollen and vacuolated, and their cristae were fragmented (Figures 8D–F). The rough endoplasmic reticulum and Golgi apparatus were distended, and the membrane-bound ribosomes were denuded. In addition, a slight deformity of the nucleus and an enlargement of the perinuclear space were observed.

A morphometric analysis revealed that the total numerical density of damaged mitochondria in the hippocampus of the OFSM group was 25.96% higher than that of the ONC group (Figure 8E). A further calculation of hippocampal CEEM revealed that energy production by the hippocampal mitochondria was reduced by 28.47% in OFSM rats compared to ONC rats (Figure 8E).

## Discussion

We investigated the effects of chronic fear stress in pregnant rats on the expression of proteins in the hippocampus of pups using iTRAQ-based proteomics analyses, which could help improve the health and the well-being of mothers and children. We found that chronic fear stress during pregnancy had a significant impact on the growth, development, and cognitive function of offspring. Similar results have been reported by Guan et al. (2016), who found that long-term maternal stress may destroy the spatial learning and memory abilities of offspring. The mechanism was determined to be an increase in corticosterone levels in maternal plasma and a decrease in hippocampal brain-derived neurotrophic factor and Arc in offspring. However, this study validated only some specific proteins and neurotrophic factors, and it remains poorly understood how chronic fear stress during pregnancy affects other proteins in offspring. The hippocampus plays an important role in early memory formation (Martin et al., 2008). Proteomics studies should be pursued to address the effects of prenatal stress on protein expression in the hippocampus of postnatal rats. In this study, pregnant rats were exposed to chronic fear stress during gestational days through observation of the electrical stimulation of male rats. iTRAQ-based proteomic and LC–MS-based metabolomic approaches and western blotting were used to elucidate changes in proteins in the hippocampus of offspring to understand the interface between maternal stress during gestational days and injury of offspring. The results showed that the DEPs that are most centrally involved affect energy metabolism and learning or memory. These findings may lead to new insights into the mechanisms of maternal stress during gestational days, leading to the development of depression and poor spatial learning and memory abilities of offspring.

Psychological stress is a common and important cause of premature ovarian failure. This is largely due to the means through which stress affects the function of the hypothalamic–pituitary–target gland axis, which leads to disorders of the hypothalamus–pituitary–ovary axis and causes premature ovarian failure (Wang X. F. et al., 2015). In addition, the Emotion-Caused Disease Theory is an important part of the etiology and the pathogenesis of the traditional system of Chinese medicine. In this study, an electric shock box was modified according to the communication box system. In this device, after the power was switched on, the male rats were shocked, causing them to scream, jump, and lose excretory control. The pregnant rats could climb up to the wires fixed on a partition to avoid the shock, but they could still hear, see, and smell what was happening to the males, which caused stress. The main advantage of this model was that it was an exclusively psychological stress model as the stimulation itself had no serious physiological effects on the pregnant rats, such as hunger, pain, or other negative stimuli, which could affect the outcome of the fetus. The commonly used animal models for chronic psychological stress include the chronic unpredictable mild stress (Zhang et al., 2018), social disruption stressor (Doherty et al., 2018), and social conflict stress (Partrick et al., 2018) models, all of which include combinations of psychological and physiological stimulation. However, diverse stimulation modes have had different effects on animals, including psychological, behavioral, learning, memory, neuroendocrine, and neurochemical effects (Katsura et al., 2002; Haleem et al., 2014). Thus, the psychological stress model in our study was ideal for a maternal stress assay. In addition, to prove the scientific reliability and rationality of the model, we evaluated it in terms of weight gain and behavior performance. The results showed that the body weight, sucrose preference, and exploratory behavior of the pregnant rats subjected to fear stress for 21 days were significantly decreased. The pregnant rats showed an apparent state of depression (Junlin et al., 2018). In previous studies, we concluded that maternal stress during gestational days resulted in behavioral changes and growth retardation in offspring. To confirm the repeatability and the reproducibility of the method and ensure experimental precision, we closely evaluated the growth and the development of offspring and their behavior. The results for birth weight, physical and reflex development, TST, OFT, and MWM in the offspring indicate that fear stress in pregnant rats could lead to developmental retardation and impairment of spatial learning and memory of offspring. In the MWM Test, not all the indexes between the two groups were significantly different, and the degree of impairment might not reach the disease state. However, it can be found that the learning and memory ability of OFSM rats was lower than that of ONC rats. These results indicate that offspring experienced an apparent state of behavioral change and growth retardation.

The hippocampus is thought to play an important role in the formation of early memories (Martin et al., 2008). Therefore, the hippocampal protein profiles of offspring were analyzed to identify potential biological relationships between prenatal stress and pathological changes in offspring. Using iTRAQ-based proteomics analyses, 158 proteins were found to exhibit a significant differential expression between the two groups. Among these, 58 were upregulated (Table 1), and 100 were downregulated (Table 2) in the hippocampus of the rats in the OFSM group relative to the ONC group, and the number of downregulated proteins was much bigger than that of the upregulated proteins. To develop the understanding of the effects of prenatal stress on the learning and memory behavior of the offspring, GO classification and pathway enrichment of the DEPs were performed. The pathway analyses showed that the DEPs mainly had functions in oxidative phosphorylation (00190), Alzheimer’s disease (05010), amoebiasis (05146), and glycolysis/gluconeogenesis (00010) (Figure 6). The GO annotations also showed that the DEPs were mainly enriched in ATP metabolism and learning or memory. These results show that the expression of many learning- and memory-related proteins was changed. Meanwhile, the DEPs related to neurodegenerative diseases and learning or memories were mainly distributed in downregulated proteins (Table 2). These results suggest that fear stress in pregnant rats might increase the risk for neurodegenerative diseases in offspring. In addition, the pathway network of the DEPs (Figure 6A) showed that the impairment of the hippocampal cGMP–PKG signaling pathway (04022), glycolysis/gluconeogenesis (00010), and the oxidative phosphorylation pathway (00190) may be the main reason for the learning and memory impairment in the offspring. The Alzheimer’s disease pathway connected with the cGMP–PKG signaling pathway, oxidative phosphorylation (00190), and glycolysis/gluconeogenesis through four DEPs, five DEPs, and one DEP, respectively. These results lead us to speculate that the impairment of learning and memory in offspring was due to the disturbance of the cGMP–PKG signaling pathway and of oxidative phosphorylation in the hippocampus. Previous studies have found that cGMP signaling dysfunction is highly associated with memory deficit. Phosphodiesterase-5 inhibitors prevent the breakdown of cGMP and then stimulate cAMP/protein kinase A/cAMP element-binding protein (CREB) phosphorylation and cGMP/PKG/CREB pathway activation to enhance synaptic transmission (Sierksma et al., 2013; Zuccarello et al., 2020). In addition, the NO-cGMP–PKG pathway is able to regulate the production of neurotransmitters, including glutamate, to mediate synaptic plasticity, which is essential for learning and memory (Garthwaite, 2008). In addition, the NO-cGMP–PKG signaling pathway regulates synaptic plasticity and fear memory consolidation by activating ERK/MAP kinase in the lateral amygdala (Ota et al., 2008). Furthermore, the CREB protein/CREB signaling pathway regulates the expression of genes that are important for synaptic plasticity (Lonze and Ginty, 2002). Therefore, we speculated that fear stress during pregnancy might contribute to learning and memory impairment in offspring, and this might be associated with the dysfunction of the cGMP/PKG signaling pathway in the hippocampus of the offspring. Further analyses of the DEPs led us to conclude that most of those in the signaling pathway tended to decrease, such as Ppp3r1, Slc25a4, Map2k1, and Slc8a2. These results indicate that fear stress in pregnant rats may downregulate the cGMP–PKG signaling pathway in the hippocampus of the offspring. The interaction network of DEPs related to learning or memory and to the cGMP–PKG pathway is shown in Figure 7B. To prove the scientific reliability of the hypothesis, the cGMP level and the expression of the PKG protein in the hippocampus of the offspring were detected by ELISA and western blotting, respectively. We found that the cGMP level and the expression of PKG protein decreased significantly in the hippocampus of the rats in the OFSM group. These results support our hypothesis. In addition, our previous experience has demonstrated that the phosphorylation of the CREB protein and the expression of Synapsin-1 in the hippocampus of the offspring significantly decrease in the maternal FSM group (*P* < 0.05) (Liping et al., 2013). These results suggest that fear stress during pregnancy inhibits the cGMP/PKG signaling pathway and leads to memory impairment in the offspring.

Furthermore, in the current study, the ultrastructures of the hippocampal neuron of the offspring were observed using electron microscopy. We observed that the synaptic structure was abnormal, the membranes of pre-axoaxonic synapses and post-axoaxonic synapses were not clear, the synaptic space disappeared, and the number of synapse vesicles was decreased. In addition, the neuronal mitochondria appeared swollen and vacuolated and their cristae were fragmented in the OFSM group. Synapse loss is observed in the early stages of Alzheimer’s disease. Synapses are sites that demand large amounts of energy and depend on high levels of adenosine triphosphate derived from the mitochondria (Tang et al., 2019). Mitochondria within synaptic structures play an important role in maintaining functional neurotransmission, and this critical biological process is regulated by energy metabolism, mitochondrial distribution, mitochondrial transport, and cellular synaptic calcium flux (Tang et al., 2019). Thus, these ultrastructural changes could influence the energy metabolism of neurons and cause the impairment of learning and memory. According to GO classification, a high number of DEPs were associated with the oxidative phosphorylation pathway. These oxidative phosphorylation disorders in hippocampal neurons could influence the ability to remember and learn. These results suggest that fear stress in pregnant rats might affect the brain energy metabolism of their offspring *via* damage of neuronal ultrastructure, thereby increasing the risk for neurodegenerative diseases in offspring. In conclusion, we used LC–MS-based metabolomic and iTRAQ-based proteomic approaches to determine whether fear stress in pregnant rats affects the hippocampal metabonomics of their offspring. We found evidence that it can destroy the spatial learning and memory abilities of offspring. The mechanisms include affecting the brain oxidative phosphorylation process and inhibiting the cGMP–PKG signaling pathway.

## Data Availability Statement

All datasets presented in this study are included in the article/supplementary material.

## Ethics Statement

The animal study was reviewed and approved by the Ethics Committee of Henan University of Chinese Medicine (Henan, China).

## Author Contributions

LY and YL conceived the study. JH, LC, and QW conducted the experiments. YL and JZ analyzed and interpreted the data. YL drafted the manuscript. X-ML and PZ revised the manuscript. YL, LY, X-HL, and ZS prepared the figures and the supplementary materials. All authors approved the final version of the manuscript.

## Conflict of Interest

The authors declare that the research was conducted in the absence of any commercial or financial relationships that could be construed as a potential conflict of interest.

## References

[B1] Abe H. Hidaka N. Kawagoe C. Odagiri K. Watanabe Y. Ikeda T. (2007). Prenatal psychological stress causes higher emotionality, depression-like behavior, and elevated activity in the hypothalamo-pituitary-adrenal axis. *Neurosci. Res.* 59 145–151. 10.1016/j.neures.2007.06.1465 17658641

[B2] Bindea G. Mlecnik B. Hackl H. Charoentong P. Tosolini M. Kirilovsky A. (2009). ClueGO: a Cytoscape plug-in to decipher functionally grouped gene ontology and pathway annotation networks. *Bioinformatics* 25 1091–1093. 10.1093/bioinformatics/btp101 19237447 PMC2666812

[B3] Chen J. Woodbury M. R. Alcorn J. Honaramooz A. (2012). Dietary supplementation of female rats with elk velvet antler improves physical and neurological development of offspring. *Evid. Based Complement. Alternat. Med.* 2012:640680. 10.1155/2012/640680 PMC3323865 22550542

[B4] Chen X. Zhu Y. Wang Z. Zhu H. Pan Q. Su S. (2016). mTORC1 alters the expression of glycolytic genes by regulating KPNA2 abundances. *J. Proteomics* 136 13–24. 10.1016/j.jprot.2016.01.021 26844761

[B5] Chiavegatto S. Oliveira C. A. Bernardi M. M. (1997). Prenatal exposure of rats to diphenhydramine: effects on physical development, open field, and gonadal hormone levels in adults. *Neurotoxicol. Teratol.* 19 511–516. 10.1016/s0892-0362(97)00045-7 9392786

[B6] Clark J. D. Gebhart G. F. Gonder J. C. Keeling M. E. Kohn D. F. (1997). Special report: the 1996 guide for the care and use of laboratory animals. *ILAR J.* 38 41–48. 11528046 10.1093/ilar.38.1.41

[B7] Doherty F. D. O’Mahony S. M. Peterson V. L. O’Sullivan O. Crispie F. Cotter P. D. (2018). Post-weaning social isolation of rats leads to long-term disruption of the gut microbiota-immune-brain axis. *Brain Behav. Immun.* 68 261–273. 10.1016/j.bbi.2017.10.024 29104061

[B8] Garthwaite J. (2008). Concepts of neural nitric oxide-mediated transmission. *Eur. J. Neurosci.* 27 2783–2802. 10.1111/j.1460-9568.2008.06285.x 18588525 PMC2610389

[B9] Geng S. Yang L. Cheng F. Zhang Z. Li J. Liu W. (2019). Gut microbiota are associated with psychological stress-induced defections in intestinal and blood-brain barriers. *Front. Microbiol.* 10:3067. 10.3389/fmicb.2019.03067 32010111 PMC6974438

[B10] Guan S. Z. Ning L. Tao N. Lian Y. L. Liu J. W. Ng T. B. (2016). Effects of maternal stress during pregnancy on learning and memory via hippocampal BDNF, Arc (Arg3.1) expression in offspring. *Environ. Toxicol. Pharmacol.* 46 158–167. 10.1016/j.etap.2016.04.012 27474832

[B11] Haleem D. J. Haque Z. Ikram H. Haleem M. A. (2014). Leptin and other hormonal responses to different stressors: relationship with stress-induced behavioral deficits. *Pakistan Vet. J.* 34 504–507.

[B12] Ivani S. Karimi I. Tabatabaei S. R. Syedmoradi L. (2016). Effects of prenatal exposure to single-wall carbon nanotubes on reproductive performance and neurodevelopment in mice. *Toxicol. Ind. Health* 32 1293–1301. 10.1177/0748233714555388 25500757

[B13] Jensen Pena C. Monk C. Champagne F. A. (2012). Epigenetic effects of prenatal stress on 11beta-hydroxysteroid dehydrogenase-2 in the placenta and fetal brain. *PLoS One* 7:e39791. 10.1371/journal.pone.0039791 22761903 PMC3383683

[B14] Junlin H. Liping Y. Haijiao W. Xinmin L. Lei C. Xianghong Z. (2018). Effects of fear impaired pregnant rats on plasma ACTH and GC content and development of offspring rats. *CJTCMP* 04 1537–1539.

[B15] Katsura M. Mohri Y. Shuto K. Tsujimura A. Ukai M. Ohkuma S. (2002). Psychological stress, but not physical stress, causes increase in diazepam binding inhibitor (DBI) mRNA expression in mouse brains. *Mol. Brain Res.* 104 103–109. 10.1016/s0169-328x(02)00219-x 12117556

[B16] Ladd C. O. Huot R. L. Thrivikraman K. V. Nemeroff C. B. Meaney M. J. Plotsky P. M. (2000). Long-term behavioral and neuroendocrine adaptations to adverse early experience. *Prog. Brain Res.* 122 81–103. 10.1016/s0079-6123(08)62132-9 10737052

[B17] Laplante D. P. Barr R. G. Brunet A. Galbaud du Fort G. Meaney M. L. Saucier J. F. (2004). Stress during pregnancy affects general intellectual and language functioning in human toddlers. *Pediatr. Res.* 56 400–410. 10.1203/01.pdr.0000136281.34035.44 15240860

[B18] Liping Y. Gai L. Haijiao W. Jianghui Z. Xinmin L. Junlin H. (2018). Effects of terror stress of the pregnant rats on emotional changes of 21-day-old neonatal offspring. *CJTCMP* 01 62–64.

[B19] Liping Y. Xinmin L. Funing Y. Lei C. Xianghong Z. SIqing C. (2013). The effect of chronic fear stress during pregnancy on the synaptic structure of neurons and the expression of p-CREB and SYN-1 in the hippocampal CA3 region of offspring rats. *Lishizhen Med. Mater. Med. Res.* 10 2567–2568.

[B20] Liu H. Lü Z. Tian C. Ouyang W. Xiong Y. You Y. (2019). [Mechanism of Shenbing decoction ? in the treatment of proteinuria in chronic kidney disease: a network pharmacology-based study]. *J. South. Med. Univ.* 39 227–234. 10.12122/j.issn.1673-4254.2019.02.16 PMC6765634 30890513

[B21] Lonze B. E. Ginty D. D. (2002). Function and regulation of CREB family transcription factors in the nervous system. *Neuron* 35 605–623. 10.1016/s0896-6273(02)00828-0 12194863

[B22] Martin B. Brenneman R. Becker K. G. Gucek M. Cole R. N. Maudsley S. (2008). iTRAQ analysis of complex proteome alterations in 3xTgAD Alzheimer’s mice: understanding the interface between physiology and disease. *PLoS One* 3:e2750. 10.1371/journal.pone.0002750 18648646 PMC2453232

[B23] Notarangelo F. M. Schwarcz R. (2016). Restraint stress during pregnancy rapidly raises kynurenic acid levels in mouse placenta and fetal brain. *Dev. Neurosci.* 38 458–468. 10.1159/000455228 28214871 PMC5462839

[B24] Ota K. T. Pierre V. J. Ploski J. E. Queen K. Schafe G. E. (2008). The NO-cGMP-PKG signaling pathway regulates synaptic plasticity and fear memory consolidation in the lateral amygdala via activation of ERK/MAP kinase. *Learn. Mem.* 15 792–805. 10.1101/lm.1114808 18832566 PMC2632793

[B25] Palmfeldt J. Henningsen K. Eriksen S. A. Muller H. K. Wiborg O. (2016). Protein biomarkers of susceptibility and resilience to stress in a rat model of depression. *Mol. Cell. Neurosci.* 74 87–95. 10.1016/j.mcn.2016.04.001 27105822

[B26] Partrick K. A. Chassaing B. Beach L. Q. McCann K. E. Gewirtz A. T. Huhman K. L. (2018). Acute and repeated exposure to social stress reduces gut microbiota diversity in Syrian hamsters. *Behav. Brain Res.* 345 39–48. 10.1016/j.bbr.2018.02.005 29474810 PMC6246037

[B27] Paukov V. S. Kazanskaya T. A. Frolov V. A. (1971). Quantitative analysis of some components of myocardial electron micrographs. *Bull. Exp. Biol. Med.* 71 469–472. 10.1007/bf00808503

[B28] Schmidt M. Braun K. Brandwein C. Rossetti A. C. Guara Ciurana S. Riva M. A. (2018). Maternal stress during pregnancy induces depressive-like behavior only in female offspring and correlates to their hippocampal Avp and Oxt receptor expression. *Behav. Brain Res.* 353 1–10. 10.1016/j.bbr.2018.06.027 29958961

[B29] Senko T. Olexova L. Mokosakova M. Krskova L. (2017). Angiotensin II enhancement during pregnancy influences the emotionality of rat offspring (*Rattus norvegicus*) in adulthood. Potential use of the rat grimace scale. *Neuro Endocrinol. Lett.* 38 117–123. 28650605

[B30] Sierksma A. S. Rutten K. Sydlik S. Rostamian S. Steinbusch H. W. van den Hove D. L. (2013). Chronic phosphodiesterase type 2 inhibition improves memory in the APPswe/PS1dE9 mouse model of Alzheimer’s disease. *Neuropharmacology* 64 124–136. 10.1016/j.neuropharm.2012.06.048 22771768

[B31] Smart J. L. Dobbing J. (1971). Vulnerability of developing brain. II. Effects of early nutritional deprivation on reflex ontogeny and development of behaviour in the rat. *Brain Res.* 28 85–95. 10.1016/0006-8993(71)90526-9 5557887

[B32] Soliani F. C. D. B. G. Cabbia R. Kümpel V. D. Batistela M. F. Almeida A. G. Yamauchi L. Jr. (2018). Unpredictable chronic prenatal stress and manifestation of generalized anxiety and panic in rat’s offspring. *Prog. Neuro Psychopharmacol. Biol. Psychiatry* 85 89–97. 10.1016/j.pnpbp.2018.03.005 29596996

[B33] Takeuchi T. Iwanaga M. Harada E. (2003). Possible regulatory mechanism of DHA-induced anti-stress reaction in rats. *Brain Res.* 964 136–143. 10.1016/s0006-8993(02)04113-6 12573522

[B34] Tang J. Oliveros A. Jang M. H. (2019). Dysfunctional mitochondrial bioenergetics and synaptic degeneration in Alzheimer disease. *Int. Neurourol. J.* 23 S5–S10. 30832462 10.5213/inj.1938036.018 PMC6433209

[B35] Wang T. Chen H. Lv K. Ji G. Zhang Y. Wang Y. (2017). iTRAQ-based proteomics analysis of hippocampus in spatial memory deficiency rats induced by simulated microgravity. *J. Proteomics* 160 64–73. 10.1016/j.jprot.2017.03.013 28341594

[B36] Wang X. F. Zhang L. Wu Q. H. Min J. X. Ma N. Luo L. C. (2015). Biological mechanisms of premature ovarian failure caused by psychological stress based on support vector regression. *Int. J. Clin. Exp. Med.* 8 21393–21399. 26885082 PMC4723927

[B37] Wang Y. Ma Y. Cheng W. Jiang H. Zhang X. Li M. (2015). Sexual differences in long-term effects of prenatal chronic mild stress on anxiety-like behavior and stress-induced regional glutamate receptor expression in rat offspring. *Int. J. Dev. Neurosci.* 41 80–91. 10.1016/j.ijdevneu.2015.01.003 25639520

[B38] Weinstock M. (2001). Alterations induced by gestational stress in brain morphology and behaviour of the offspring. *Prog. Neurobiol.* 65 427–451. 10.1016/s0301-0082(01)00018-1 11689280

[B39] Weinstock M. (2017). Prenatal stressors in rodents: effects on behavior. *Neurobiol. Stress* 6 3–13. 10.1016/j.ynstr.2016.08.004 28229104 PMC5314420

[B40] Welberg L. A. Seckl J. R. (2001). Prenatal stress, glucocorticoids and the programming of the brain. *J. Neuroendocrinol.* 13 113–128. 10.1111/j.1365-2826.2001.00601.x 11168837

[B41] Wu S. Gao Q. Zhao P. Gao Y. Xi Y. Wang X. (2016). Sulforaphane produces antidepressant- and anxiolytic-like effects in adult mice. *Behav. Brain Res.* 301 55–62. 10.1016/j.bbr.2015.12.030 26721468

[B42] Xinmin L. Liping Y. Haijiao W. Jianghui Z. Junlin H. Xianghong Z. (2017). Correlation between cognitive development and levels of dopamine and 3, 4-dihydroxyphenylacetic acid in the hippocampus in 80-day-old neonatal rats born of fear-impaired pregnant rats. *Chinese J. Comp. Med.* 11 10–14.

[B43] Xu M. Shi J. Min Z. Zhu H. Sun W. (2019). A network pharmacology approach to uncover the molecular mechanisms of herbal formula kang-bai-ling for treatment of vitiligo. *Evid. Based Complement. Alternat. Med.* 2019:3053458. 10.1155/2019/3053458 PMC6875403 31781265

[B44] Youssef N. A. Lockwood L. Su S. Hao G. Rutten B. P. F. (2018). The effects of trauma, with or without PTSD, on the transgenerational DNA methylation alterations in human offsprings. *Brain Sci.* 8:83. 10.3390/brainsci8050083 29738444 PMC5977074

[B45] Zhang Y. Yuan S. Pu J. Yang L. Zhou X. Liu L. (2018). Integrated metabolomics and proteomics analysis of hippocampus in a rat model of depression. *Neuroscience* 371 207–220. 10.1016/j.neuroscience.2017.12.001 29237567

[B46] Zuccarello E. Acquarone E. Calcagno E. Argyrousi E. K. Deng S. X. Landry D. W. (2020). Development of novel phosphodiesterase 5 inhibitors for the therapy of alzheimer’s disease. *Biochem. Pharmacol.* 176:113818. 10.1016/j.bcp.2020.113818 PMC7263960 31978378

